# Differences in various biochemical and clinical parameters with respect to family history of Non Communicable Diseases in fourth year MBBS students of Karachi, Pakistan

**DOI:** 10.12669/pjms.314.7021

**Published:** 2015

**Authors:** Khalid Abdul Basit, Asher Fawwad, Muhammad Asadullah Munir, Iftikhar Ahmed Siddiqui, Sidra Siddiqui, Abdul Basit

**Affiliations:** 1 Khalid Abdul Basit, Student, MBBS, Dow University of Health Sciences, Karachi, Pakistan; 2 Asher Fawwad, M.phil. Assistant Prof., Senior Research Scientist, Research Department, Baqai Medical University, Baqai Institute of Diabetology and Endocrinology, Plot No. 1-2, II-B, Nazimabad No.2, Karachi-74600, Pakistan; 3 Muhammad Asadullah Munir, Student, MBBS, Dow University of Health Sciences, Karachi, Pakistan; 4 Iftikhar Ahmed Siddiqui, PhD. Chairman & Professor of Biochemistry, Department of Biochemistry, Baqai Medical University, Karachi, Pakistan; 5 Sidra Siddiqui, M.Sc. Statistician, Research Department, Baqai Medical University, Baqai Institute of Diabetology and Endocrinology, Plot No. 1-2, II-B, Nazimabad No.2, Karachi-74600, Pakistan; 6 Abdul Basit, F.R.C.P. Professor of Medicine, Department of Medicine, Baqai Medical University, Baqai Institute of Diabetology and Endocrinology, Plot No. 1-2, II-B, Nazimabad No.2, Karachi-74600, Pakistan

**Keywords:** Leptin, Renin, Medical Student, Non-communicable disease

## Abstract

**Objective:**

To observe the differences of various biochemical and clinical parameters with respect to Family History (FH) of Non-communicable Diseases (NCDs) in fourth year Bachelor of Medicine, Bachelor of Surgery (MBBS) students.

**Methods:**

This observational study was conducted at Baqai Institute of Diabetology & Endocrinology from December 2013 to January 2014. Total 50 medical students from Dow University of Health Sciences (DUHS) participated in the study. Statistical Package for Social Sciences (SPSS) version 13 was used to analyze the data. For cross tabulation and mean comparison z-test and t test were applied.

**Results:**

Out of 50 subjects, there were 26 (52%) females. Mean age of the study population was 21.56 ± 0.90 years. Mean serum cholesterol levels with positive FH of NCDs was significantly higher than negative FH of NCDs (p=0.005). Mean value of low density lipoprotein (LDL) of positive family history of NCDs was found higher than those with negative FH (p=0.006) being statistically significant. The insulin levels in subjects with positive FH of NCDs were higher than subjects with negative FH of NCDs (p=0.685). However, serum leptin and plasma renin showed no significant difference with the negative FH of NCDs being higher compared to positive FH of NCDs (p=0.068) and (p=0.884) respectively. However, Waist circumference, Body mass index and central obesity in subjects with positive FH of NCDs shows increasing trend but no statistically significant difference (p > 0.05) was observed.

**Conclusion:**

In our study of various biochemical and clinical parameters with respect to FH of NCDs, Serum Cholesterol and LDL levels were observed higher and statistically significant.

## INTRODUCTION

Diabetes Mellitus (DM) and hypertension are interrelated disorders coexisting and attributing as risk factors for cardiovascular disease.^1^ DM is influenced by an elevated body mass index (BMI) and hypertension is comorbidity for developing DM.^2^ The risk of macro vascular complications of DM which includes stroke, coronary artery disease, heart failure and myocardial infarction is also associated with hypertension.^3^

One fourth of the world’s population had metabolic syndrome and is at increased risk of cardiovascular events.^4^ The increasing burden of Non-communicable disease (NCDs) in developing countries is leading to 80% of NCD deaths worldwide.^5^

Leptin a product of obese gene secreted mainly by white adipocytes, is positively correlated with BMI and total body fat with linear relationship in obese patients.^6^ Leptin resistance is pathognomonic mechanism rather than leptin deficiency.^7^ An elevation in leptin levels is consistent with hypertension, insulin resistance, obesity, Polycystic Ovary Syndrome (PCOS) and type 2 diabetes.^8^

Renin, a proteolytic enzyme, mainly secreted by the juxtaglomerular cells in the kidney in response to renal hypoperfusion, systemic hypotension, low plasma sodium levels and increased sympathetic outflow helps regulation of normal blood pressure and sodium homeostasis.^9^ Plasma renin activity among the hypertensive patients may be elevated; normal or decreased.^10^ Essential hypertensive patients with high PRA are at greater risk of developing stroke, cardiovascular disease (CVD) and renal complications.^11^

We conducted this study to observe the differences of various biochemical and clinical parameters with respect to Family History (FH) of Non-communicable Diseases (NCDs) in fourth year Bachelor of Medicine, Bachelor of Surgery (MBBS) students.

## METHODS

The observational study was conducted at Baqai Institute of Diabetology & Endocrinology (BIDE) including 50 students of Dow University of Health Sciences (DUHS) Karachi, from December 2013 to January 2014. We focused on patients with type 2 diabetes, hypertension and CVD for NCDs in this study.

### Inclusion criteria

Non diabetic, non-hypertensive healthy students (males and females), aged 20 - 25 years were eligible for the study.

### Exclusion criteria

The students with any comorbid condition like hypertension, diabetes, renal disease, liver disease, pulmonary tuberculosis (TB), Poly cystic disease, metabolic syndrome and CVD were excluded to rule out any derangements in the laboratory values.

The ethical approval was obtained from the Institutional Review Board (IRB) of BIDE. The participants were briefed about the study and informed consent was taken. An interview based Performa (demographic data along with age, height, weight, waist hip circumference, systolic and diastolic pressure, medical history, family history and drug history) and physical examination was conducted. Weight, height (to the nearest of 0.1cm and 0.1 kg) and a resting state blood pressure was measured. An adult footed mercury sphygmomanometer was used on brachial artery of hand with a 5 minutes delay in recording the reading. A mean value of two readings was used. Hypertension was defined as blood pressure ≥140/90 mmHg.^12^ Twelve hours peripheral fasting blood samples were collected for the estimation of biochemical parameters. Samples of clotted blood were centrifuged to separate serum for analysis. EDTA plasma was separated for plasma renin estimation.

Fasting blood glucose, serum lipid profile, magnesium and uric acid levels were measured with the enzymatic colorimetric method (Selectra Pro S, ELItech, France).^13^ Serum leptin, plasma renin and serum insulin were determined by Enzyme-Linked Immunosorbent Assay (ELISA) method. Association of serum leptin and plasma renin with FH of NCDs, specifically the diabetes and hypertension was observed. All test were performed in BIDE clinical and research laboratory.

### Statistical Analysis

Statistical Package for Social Sciences (SPSS) version 13.0 was used for data analysis. The continuous variables i.e. Age, Weight, Height, Waist circumference, Body mass index (BMI), Systolic and Diastolic blood pressure, Fasting plasma glucose, Serum Cholesterol, Serum Triglycerides, High Density Lipoprotein (HDL), Low Density Lipoprotein (LDL), Serum Leptin, Plasma Renin, Insulin and HOMA IR were presented as Mean ± SD. T-test and z-test were applied (p<0.05 statistically significant).

## RESULTS

Baseline clinical and biochemical characteristics of the study population gender wise are shown in Table-I. The mean age of study population was (21.56 ± 0.90) years, BMI (20.36 ± 2.83), mean weight (58.97 ± 11.88) kg and mean waist circumference was (80.12 ± 17.90) cm. Mean values of plasma renin and serum leptin of the study population were found to be 22.12 ± 24.21 and 2.60 ± 1.09 respectively. A significant difference was observed in the obese male subjects as compared to female (p=0.007). No such significant difference was found in normal and overweight.

**Table-I:** Baseline characteristics of clinical and biochemical parameters of the study population.

	Male n=24	Female n=26	p-value	Overall n=50
Age (years)	21.46 ± 1.14	21.65 ± 0.62	0.452	21.56 ± 0.90
Systolic blood pressure (mmHg)	119.16 ± 8.42	102.88 ± 10.50	<0.0001	110.70 ± 12.53
Diastolic blood pressure (mmHg)	81.58 ± 8.05	69.50 ± 9.10	<0.0001	75.30 ± 10.48
Weight (kg)	64.70 ± 13.08	53.67 ± 7.63	0.001	58.97 ± 11.88
Height (cm)	174.13 ± 7.66	161.55 ± 6.14	<0.0001	167.59 ± 9.33
Waist circumference (cm)	83.71 ± 9.03	76.94 ± 22.82	0.190	80.12 ± 17.90
Hip circumference (cm)	91.85 ± 12.39	95.57 ± 13.14	0.321	93.87 ± 12.81
Body mass index (kg/m^2^)	19.47 ± 3.63	20.61 ± 2.64	0.439	20.36 ± 2.83
Fasting blood sugar (mg/dl)	78.95 ± 6.40	75.03 ± 5.27	0.022	76.92 ± 6.11
Serum cholesterol (mg/dl)	154.45 ± 24.80	159.03 ± 23.87	0.509	156.84 ± 24.18
Serum triglyceride (mg/dl)	87.95 ± 45.79	68.38 ± 27.60	0.071	77.78 ± 38.34
High density lipoprotein (mg/dl)	44.45 ± 7.41	57.57 ± 13.25	<0.0001	51.28 ± 12.62
Low density lipoprotein (mg/dl)	82.79 ± 27.06	85.15 ± 23.19	0.741	84.02 ± 24.89
Serum Creatinine (mg/dl)	0.98 ± 0.13	0.83 ± 0.06	<0.0001	0.90 ± 0.12
Serum Leptin (ng/ml)	2.01 ± 0.58	3.15 ± 1.18	<0.0001	2.60 ± 1.09
Plasma Renin (pg/ml)	18.01 ± 24.25	25.90 ± 24.00	0.253	22.11 ± 24.21
Insulin level (uIU/ml)	12.67 ± 5.32	8.87 ± 3.41	0.004	10.70 ± 4.79
HOMA IR	2.49 ± 1.15	1.65 ± 0.66	0.002	2.05 ± 1.01
Central Obesity (%)				
Normal	47.2	52.8	0.637	100.0
Abnormal	50.0	50.0	> 0.999	100.0
Body mass index (%)				
Normal weight	43.2	56.8	0.245	100.0
Over weight	33.3	66.7	0.248	100.0
Obese	85.7	14.3	0.007	100.0

Data presented as Mean ± SD, P < 0.05 considered as statistically significant.

Comparison in percentages: p-value calculated by z-test.

Table-II shows the comparison of FH of NCDs for the continuous variables. Statistically significant difference was observed in the mean value of age (21.78 ± 0.65 vs. 21.17 ± 1.15, p=0.020). Students with positive FH of NCDs had cholesterol significantly higher than those with negative FH (163.87 ± 24.16 vs. 144.33 ± 19.03, p=0.005). LDL mean values in students with positive FH of NCDs increased significantly than those with negative FH (91.12 ± 23.52 vs. 71.38 ± 22.66, p=0.006). The findings of serum leptin and plasma renin showed no significant difference as negative FH of NCDs was slightly higher than the positive FH of NCDs (2.39 ± 0.93 vs. 2.98 ± 1.27, p=0.068) and (21.73 ± 25.88 vs. 22.79 ± 21.60, p=0.884) respectively. Insulin levels were slightly higher in positive FH of NCDs than in negative FH of NCDs (10.90 ± 4.68 vs. 10.32 ± 5.09, p=0.685).

**Table-II:** Comparison of Family history of NCDs.

	Positive FH of NCDs n=32	Negative FH of NCDs n=18	p-value	Overall n=50
Age (years)	21.78 ± 0.65	21.17 ± 1.15	0.020	21.56 ± 0.907
Systolic blood pressure (mmHg)	110.62 ± 12.87	110.83 ± 12.27	0.956	110.70 ± 12.53
Diastolic blood pressure (mmHg)	74.68 ± 10.87	76.38 ± 9.97	0.587	75.30 ± 10.48
Weight (kg)	59.68 ± 13.49	57.69 ± 8.50	0.574	58.97 ± 11.88
Height (cm)	166.87 ± 9.76	168.86 ± 8.64	0.475	167.59 ± 9.33
Waist circumference (cm)	81.37 ± 21.13	77.76 ± 9.30	0.508	80.12 ± 17.90
Hip circumference (cm)	93.89 ± 15.06	93.82 ± 7.52	0.986	93.87 ± 12.81
Body mass index (kg/m^2)^	21.15 ± 2.73	19.13 ± 2.67	0.095	20.36 ± 2.83
Fasting blood sugar (mg/dl)	76.31 ± 6.23	78.00 ± 5.90	0.354	76.92 ± 6.11
Serum cholesterol (mg/dl)	163.87 ± 24.16	144.33 ± 19.03	0.005	156.84± 24.18
Serum triglyceride (mg/dl)	78.31 ± 38.36	76.83 ± 39.41	0.897	77.78 ± 38.34
High density lipoprotein (mg/dl)	53.12 ± 13.82	48.00 ± 9.62	0.171	51.28 ± 12.62
Low density lipoprotein (mg/dl)	91.12 ± 23.52	71.38 ± 22.66	0.006	84.02 ± 24.89
Serum creatinine (mg/dl)	0.89 ± 0.13	0.91 ± 0.10	0.604	0.90 ± 0.12
Serum Leptin (ng/ml)	2.39 ± 0.93	2.98 ± 1.27	0.068	2.60 ± 1.09
Plasma Renin (pg/ml)	21.73 ± 25.88	22.79 ± 21.60	0.884	22.11 ± 24.21
Insulin levels (uIU/ml)	10.90 ± 4.68	10.32 ± 5.09	0.685	10.70 ± 4.79
HOMA IR	2.08 ± 0.99	2.00 ± 1.07	0.787	2.05 ± 1.01
Central Obesity (%)				
Male normal	62.5	37.5	0.157	100.0
Male abnormal	71.4	28.6	0.108	100.0
Female normal	63.2	36.8	0.104	100.0
Female abnormal	71.4	28.6	0.108	100.0
Body mass index (%)				
Normal weight	59.5	40.5	0.103	100.0
Over weight	66.7	33.3	0.248	100.0
Obese	85.7	14.3	0.007	100.0

Data presented as Mean ± SD, P < 0.05 considered as statistically significant

Comparison in percentages: p-value calculated by z-test. FH: Family History, NCDs: Non-communicable Diseases.

Central obesity showed no significant difference in the of positive FH of NCDs and negative FH of NCDs of male and female both (p>0.05). In positive FH of NCDs, the percentage of obese were higher than in negative FH of NCDs. Normal and overweight percentages were also higher in the positive FH of NCDs than negative FH of NCDs but the statistical significance is not met.

## DISCUSSION

We have found higher association of positive FH of NCDs particularly CVD with BMI, BP and LDL. Our results showed that FH of hypertension have higher association with total cholesterol. Literature search sowed that in a study of BRICS (Brazil, Russia, India, China, and South Africa) countries, association of obesity with hypertension and diabetes in all the four countries was studied.^14^ Similarly a study on Chinese steelworkers showed significant association of fatal CVD with hypercholesterolemia.^15^ In Estonian adult population study, a decrease in high density lipoprotein (HDL) cholesterol and increase in triglyceride were associated with increased probability of hypertension.^16^ In study of a large, multi-ethnic population-based cohort from sites across the United States, higher leptin concentrations were associated with hypertension.^17^

Yet another study showed an inverse relationship between risk factors control and BMI.^18^ Another study showed a significantly higher BMI in the high blood pressure group and increased levels of LDL cholesterol.^19^ A study of Korean adolescents had BMI correlated positively with Systolic Blood Pressure in the normal weight and overweight groups. High BMI and high waist circumference (WC) significantly increased the incidence of high Systolic Blood Pressure.^20^

A higher association of HDL, TG, Leptin & Renin was seen with having FH of diabetes in our study. In a patient control study, higher concentrations of total cholesterol and triglycerides and lower HDL cholesterol were noted in diabetics as compare to controls.^21^ In another study dyslipidaemia was elevated LDL-C in type 2 diabetes (T2DM).^19^ Low HDL cholesterol, high LDL cholesterol, hypercholesterolaemia and hypertriglyceridemia were found in T2DM group of subjects.^4^ Clinical and biochemical parameters are important tool for the prediction of high risk population with respect to NCDs.
